# Evaluating Past Range Shifts and Niche Dynamics of Giant Pandas Since the Last Interglacial

**DOI:** 10.3390/ani15060801

**Published:** 2025-03-12

**Authors:** Yadong Xu, Xiaoan Liu, Aimei Yang, Ziyi Hao, Xuening Li, Dan Li, Xiaoping Yu, Xinping Ye

**Affiliations:** 1 College of Life Sciences, Shaanxi Normal University, Xi’an 710119, China; yadong@snnu.edu.cn (Y.X.);; 2 Research Center for UAV Remote Sensing, Shaanxi Normal University, Xi’an 710062, China; 3 Changqing Field Station for Ecological Research & Education, Shaanxi Normal University, Xi’an 710119, China; 4 Shaanxi Academy of Forestry Research Center for the Qinling Giant Panda, Xi’an 710100, China; 5 Shaanxi Provincial Field Observation & Research Station for Golden Monkey, Giant Panda and Biodiversity, Xi’an 710032, China

**Keywords:** range shift, climatic niche, Paleo-climate change, ecological niche model, ecospat, WorldClim, *Ailuropoda melanoleuca*

## Abstract

Understanding the response of species to past climate change provides great opportunities to know their adaptive capacity for resilience under future climate change. Since the Late Pleistocene, dramatic climate fluctuations have significantly impacted the distribution of giant pandas (*Ailuropoda melanoleuca*). However, how the spatial distribution and climatic niche of giant pandas shifted in response to past climate change remain poorly understood. Based on the known distribution records (fossil and present day) and the most updated climate projections for the Last Interglacial (LIG; ~120 ka), Last Glacial Maximum (LGM; ~22 ka), Mid-Holocene (MH; ~6 ka), and the present day, we predicted and compared the distribution and climatic niche of giant pandas. The results show that giant pandas have undergone a considerable range contraction (a 28.27% reduction) followed by a marked range expansion (a 75.8% increase) during the LIG–LGM–MH period, while its climatic niche remained relatively stable. However, from the MH to the current, both the distribution area and climatic niche of giant pandas have undergone significant changes. Our findings suggest that the giant panda may adjust its distribution to track stable climatic niches in response to future climate change. Future conservation planning should designate accessible areas for giant pandas and adjust priority conservation areas as needed.

## Simple Summary

Understanding how species respond to climate change is essential for conservation. Giant pandas, an iconic species endemic to China, have experienced significant climate fluctuations since the Late Pleistocene, impacting their distribution. This study analyzed the distribution and climatic niche of giant pandas during major climate periods, including the Last Interglacial, Last Glacial Maximum, Mid-Holocene, and present day. Results reveal that while giant pandas faced range contractions and expansions, their climatic niche remained relatively stable, suggesting niche conservatism. However, since the Mid-Holocene, both their distribution and climatic niche have significantly changed, with their current niche representing only a fraction of their historical range. This indicates that giant pandas can adapt to future climate changes by shifting their distribution to track suitable climatic niches. Conservation efforts should focus on habitat connectivity, regularly adjusting priority areas to mitigate the impacts of ongoing climate change. This study provides valuable insights for ensuring the long-term survival of giant pandas under future climate scenarios.

## 1. Introduction

Rapid climate change, often defined as changes occurring over decades to centuries [1], is having a profound impact on global biodiversity by driving continuous alterations in ecosystems, including shifts in natural landscapes, vegetation cover, and climate zones [2,3,4,5,6]. These changes have disrupted many species’ habitats [7], living conditions [8,9], and biological interactions [10,11], thereby exacerbating the risk of species extinction [12]. Different species employ different strategies to cope with climate change. Some species have altered their ranges to track suitable natural habitats, such as some birds and stream fishes [13,14,15]. In such cases, species change their geographic distribution, but their ecological niche may remain stable in the climatic space, i.e., niche conservatism [15,16,17]. There are also some species that can adjust their climatic niches over time without needing to relocate to new areas for survival, such as bushy-tailed woodrat (*Neotoma cinerea*) [18]. Understanding how species responded to past climate fluctuations is essential for anticipating future responses and developing informed conservation strategies under ongoing rapid climate change.

Investigating a species’ historical distribution and climatic niche dynamics provides key insights into how species have adapted to climate variability, which is crucial for managing endangered species under current and future climatic conditions [19,20]. Such studies can reveal whether species have maintained their original habitats or shifted to new ones [18,21]. Furthermore, they help assess whether species exhibit niche conservatism—maintaining stable ecological requirements over time—or adapt to climate change by altering their niche [14,17,18]. Such insights are critical for identifying species at higher risk of extinction and for developing targeted conservation strategies [22,23]. The rich fossil record, combined with advances in radiocarbon and uranium-series dating techniques [24,25], enables the reconstruction of species’ distributions during specific historical periods, providing a foundation for present-day conservation decision-making.

The ecological niche model (ENM) defines a species’ ecological requirements within environmental, climatic, or niche-space dimensions, while the species distribution model (SDM) projects these ecological preferences onto geographic space to predict potential distributions [26,27]. They are currently widely used in fields such as predicting species distribution [28], constructing ecological niches [29], plant introduction [30], and biological invasion [31]. In addition, climate fluctuations since the Late Pleistocene (~126 ka) can be used as a virtual laboratory to understand the ecological niches of species under different climatic conditions. New geophysical research indicates that dramatic climate shifts during the past occurred over just a few years, possibly at a faster rate than current changes [1,32]. These rapid transitions, particularly during the Last Interglacial (LIG; ~120 ka); Last Glacial Maximum (LGM; ~22 ka); and Mid-Holocene (MH; ~6 ka), significantly influenced species distribution, leading to population expansions and bottlenecks [33,34,35]. Compared to the present day, the LIG had a warmer global climate and a higher sea level of 4–8 m [36]. During the LGM, glaciers reached their maximum extent, and most regions of the world experienced extreme cold and aridity [37,38]. In contrast, the MH was characterized by a relatively stable, warm, and humid global climate [39,40,41]. The significant climate fluctuations during these periods and the availability of easily accessible climate data provide a solid foundation for studying the climate niche dynamics of species.

Giant pandas (*Ailuropoda melanoleuca*) are a large mammal and have received much attention and research for their distinctive appearance and ecological habits [42,43,44,45]. Fossil evidence suggests that this species was once widely distributed throughout much of southern China [46,47,48]. Due to paleo-climatic changes and massive human expansion, it is currently distributed only in six mountainous regions (Qinling, Minshan, Qionglai, Daxiangling, Xiaoxiangling, and Liangshan) on the eastern edge of the Tibetan Plateau [45,49]. Their unique evolutionary trajectory spanning 8 million years and specialized bamboo-dependent habitat requirements make them particularly suitable for studying climate change impacts [45,50]. Existing research suggests that current climate change will significantly increase the risk of extinction for giant pandas, with some studies predicting that they could lose more than 60% of their suitable habitat by the end of the century [7,51,52,53]. However, it is important to note that giant pandas have survived multiple periods of rapid climatic shifts throughout their evolutionary history [35]. Their ability to endure such changes raises the possibility that the projected threat posed by contemporary climate change may be overstated [1,32]. Understanding the distribution range and climatic niche of giant pandas during past climate fluctuations can provide valuable insights into their capacity for resilience in the face of ongoing climate change. Such knowledge is crucial for developing effective conservation strategies that are grounded in a nuanced understanding of both historical and current environmental pressures.

Therefore, this study aims to investigate the potential distribution and climatic niche dynamics of the giant panda during significant climate fluctuations in the Late Pleistocene and Holocene. Specifically, we address the following three research questions: (1) How did the potential geographic distribution of giant pandas change during past climate change periods, such as the LIG, LGM, and MH? (2) Did the giant panda exhibit climatic niche conservatism, or did its climatic niche shift in response to historical climate changes? (3) What are the implications of the historical distribution and climatic niche dynamics of giant pandas for their current and future conservation under ongoing rapid climate change? By addressing these questions, we aim to enhance the understanding of the giant panda’s resilience to climate change and to inform effective conservation strategies.

## 2. Materials and Methods

### 2.1. Study Area and Occurrence Data

**Figure 1:**
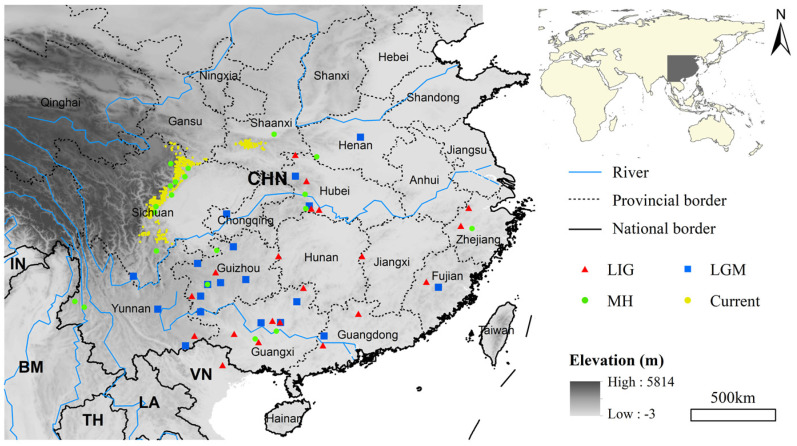
Map of the study area with a digital elevation map in the background. Red triangles, blue squares, and green filled circles represent the fossil points used for modelling during the Last Interglacial (LIG), Last Glacial Maximum (LGM), and Mid-Holocene (MH), respectively. The yellow solid circles represent the occurrence records used for modelling in the current era.

The study area encompasses all known fossil records of giant pandas and represents the species’ largest geographical distribution across different periods (18~41° N, 94.75~123° E), including most of China as well as northern Myanmar, Thailand, Laos, and Vietnam (Figure 1) [43,46,54]. The total area encompasses about 5.33 million square kilometers, with an elevation of −3 to 5814 m.

Occurrence data for giant pandas in the current era are from the Third National Giant Panda Survey (1999–2003) [55]. This study filtered the data to ensure that each grid retained at most one data point, resulting in a total of 328 data points for subsequent modelling (Figure 1). A total of 191 fossil records (duplicates excluded) for giant pandas were obtained from the Database of Vertebrate Paleontology and Paleoanthropology, the Paleobiology Database (https://paleobiodb.org, accessed on 8 March 2023), as well as from the published literature from 1974 to 2018 [47,48,54,56,57,58]. These fossil records have well-defined geological ages, with 60 of them further providing relatively specific radiocarbon dating or uranium-series dating information, accounting for approximately 31.94% of the total fossil records. The dating of fossils typically involves a certain degree of uncertainty [59]. In this study, a relatively broad time range (±10,000 years) was chosen to accommodate measurement errors, avoiding the problem of insufficient sample size caused by overly strict time constraints, which could affect the reliability of the model. Subsequently, we used the geological age information from fossil records, and those described as early the Late Pleistocene were assigned to LIG, those described as the Late Pleistocene end were assigned to LGM, and those described as Holocene were assigned to MH. Consequently, we obtained 20 fossil records from the LIG period, 20 from the LGM period, and 20 from the MH period (Figure 1). Additionally, there were 28 fossil records with uncertain specific dating information within the Late Pleistocene, which were utilized to verify the accuracy of predictions. The remaining 103 fossil records were excluded from the analysis. For fossil records without latitude and longitude information, we used the coordinate picking system of Baidu Maps to obtain data based on the fossil location description (https://api.map.baidu.com/lbsapi/getpoint/index.html, accessed on 20 September 2021).

### 2.2. Climate Variables

Nineteen climate variables for each period were downloaded from the WorldClim database (https://www.worldclim.org, accessed on 28 September 2021) (Table 1). The climate data for the LGM and MH periods were derived from the weighted average of three general circulation models (GCMs), the Model for Interdisciplinary Research on Climate–Earth System Model (MIROC-ESM), the Community Climate System Model Version 4 (CCSM4), and the Max Planck Institute–Earth System Model (MPI-ESM-P), with equal weights assigned to each model [60,61,62]. For the LIG and the current period, only one set of climate data was involved, so no general circulation models were considered. The minimum resolution of climate data for the LGM in the WorldClim dataset is 2.5 arc minutes. Therefore, we opted for a spatial scale of 2.5 arc minutes for this study. The original climate data for the LIG were collected at a higher resolution of 30 arc seconds. To ensure consistency in the analysis, we resampled the raw data to a resolution of 2.5 arc minutes using bilinear interpolation [63].

Multicollinearity poses significant challenges in ecological data analysis, potentially leading to severe statistical distortions [64]. To address this, we applied Pearson’s correlation coefficient to assess relationships between variables, considering those with |r| > 0.7 as highly correlated [65]. We also employed the variance inflation factor (VIF) to detect and quantify multicollinearity among predictors [65,66,67]. Through an iterative process of removing correlated variables and recalculating VIF, we retained only those variables with VIF values below 5, ensuring minimal multicollinearity within the dataset [65]. This procedure was conducted using the ’fuzzySim’ package in R version 4.0.2 [68,69]. As a result, seven variables were ultimately selected from the original set of nineteen for modelling.

### 2.3. Model Construction and Optimization

We utilized MaxEnt 3.4.1 to construct ecological niche models using fossil records from four periods (LIG, LGM, MH, and current) and seven selected climate variables (Table 1). The maximum entropy model is built by selecting the model with greatest uncertainty that satisfies given constraints [70]. Compared to other ecological niche models, MaxEnt has been shown to produce robust predictions with small sample sizes (fifteen is sufficient) [71,72,73]. During the modelling process, 75% of the occurrence records were randomly selected as training data, while 25% were used for testing. The output format was set to Cloglog. In cases where occurrence points are limited, using the subsample method with 10 replicates is a reasonable approach to reduce bias and improve the robustness of the model. The subsample method allows the data to be split into multiple subsets, ensuring that each subset is used both for training and validation [74,75]. This approach helps mitigate the risk of overfitting and allows for a more comprehensive evaluation of the model’s performance [74]. The Jackknife analysis was employed to assess the relative contribution of each climate variable, and the most important variables were identified based on their percentage contribution. Additionally, generalized additive models (GAMs) were used to generate response curves showing the relationship between the probability of giant panda occurrence and climate variables across different periods.

We used the ‘ENMeval v0.3.1’ package to optimize the MaxEnt model, employing the Checkerboard 2 method to divide the study area into four bins [76]. This masked geographic structure method helps better adjust the model’s regularization level. The MaxEnt model’s regularization level consists of two parameters: the regularization multipliers (RMs) and the feature classes (FCs). Specific combinations of these parameters provide better results than the default settings, especially when dealing with small sample sizes [77,78,79,80]. MaxEnt can use five types of features, including linear (L), quadratic (Q), product (P), hinge (H), and threshold (T) features. In this study, to optimize the MaxEnt model for four periods, the RM was set to range from 0.5 to 4, increasing by 0.5 for a total of eight levels. Meanwhile, six feature combinations were used: L; L and Q; H; L, Q, and H; L, Q, H, and P; and L, Q, H, P, and T. In total, 48 parameter combinations were calculated for each period based on these permutations and combinations. We tested each model’s complexity using the 48 parameter combinations, evaluating them based on four metrics: the area under the receiver–operating characteristic curve for test localities (AUC_TEST_), the difference between training and testing AUC (AUC_DIFF_), the 10% omission rate for test localities (OR_10_), and the Akaike information criterion corrected for small sample sizes (AICc) [81,82,83,84,85]. A higher AUC_TEST_, closer to 1, reflects a model’s better ability to discriminate between conditions at testing localities [83,84]. Lower AUC_DIFF_ and OR_10_ values indicate less overfitting, and the model with the lowest AICc value (i.e., ΔAICc = 0) was considered the best among the parameter sets tested [81,83,84,85].

### 2.4. Spatial Analyses

The threshold selection method based on maximizing the sum of sensitivity and specificity (MaxSSS) is a promising approach, especially when presence-only data are used in the study [86,87]. Cells with predicted probability of occurrence values below the MaxSSS threshold were assigned a value of 0, indicating an unsuitable habitat. Areas with values above the MaxSSS threshold represent the potential distribution range for pandas during the given period. The SDM Toolbox 2.4 in ArcGIS 10.2 was used to calculate the centroid of the suitable distribution area for each time period, which is the geographic center of a region [88]. Centroid shifts were used to describe the direction and distance of the pandas’ range shift in three time periods (LIG to LGM, LGM to MH, and MH to current).

### 2.5. Comparison of Climatic Niches

We used the ‘ecospat v3.2’ package in R (version 4.0.2) to compare the climatic niches of giant pandas across different time periods (LIG, LGM, MH, and current) [89]. The framework applies the kernel smoother to the species occurrence density in the environmental gridded space to quantify ecological niche overlap and test for ecological niche equivalence and similarity [90]. First, a gridded climatic ecological niche space for giant pandas was defined based on the first two axes of principal component analysis (PCA) built on the four aforementioned climatic variables. Then, 10,000 points were randomly selected in the potential distribution of pandas in each period and were transformed into occurrence densities corresponding to environmental values by a kernel smoother. Secondly, Schoener’s D values were calculated to quantify climatic ecological niche overlap at different time periods, which varies from 0 (no overlap) to 1 (complete overlap). Finally, a niche equivalency (assessing if niches are statistically indistinguishable through niche identity permutations) and niche similarity test (evaluating directional similarity relative to environmental backgrounds) with 95% confidence intervals were conducted to determine whether the climatic ecological niche of giant pandas is more similar than expected by chance in the two time periods [90,91].

## 3. Results

### 3.1. Model Optimization and Accuracy Evaluation

**Figure 2:**
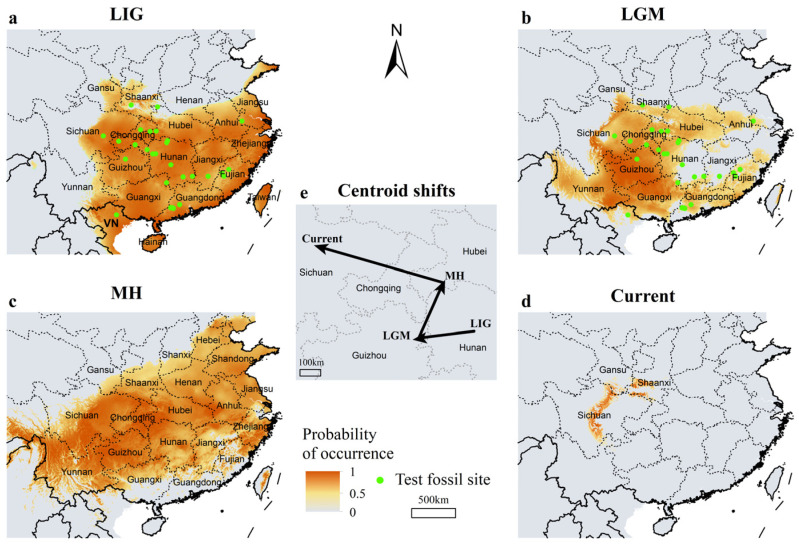
The range of giant pandas during the Last Interglacial (**a**), the Last Glacial Maximum (**b**), the Mid-Holocene (**c**), and current period (**d**), and range shifts since the Last Interglacial (**e**). The direction and length of the arrows represent the direction and distance of shift. The green solid circles indicate undated giant panda fossils since the LIG.

The results of model optimization show that the optimal model for the LIG period has a feature class of Linear-Quadratic-Hinge-Product (LQHP) and a regularization multiplier of 1.5, with a ΔAICc of 0, indicating the best model performance (Table 2). For the LGM, MH, and current periods, the optimal models (ΔAICc = 0) had feature classes set to Linear-Quadratic-Hinge (LQH), with regularization multipliers of 2, 4, and 0.5, respectively (Table 2). Among the optimized models for the four periods, the lowest AUC_TEST_ value was observed for the MH period (0.890), while the current period reached 0.998. All AUC_DIFF_ values were less than 0.05, and OR_10_ values were below 0.3, indicating that the optimized models exhibited better predictive performance compared to the default settings (Table 1). Of the undated giant panda fossils since the LIG, all are located within the predicted suitable range for the LIG period (Figure 2a), and the predicted range for the LGM period covers 78.57% (Figure 2b). This indicates that our predictions are highly accurate.

### 3.2. Range Shifts of Giant Pandas Since the Last Interglacial

Model results indicate that during the LIG, giant pandas were widely distributed in southern China (Figure 2a), covering approximately 2.20 million km^2^, which accounted for 23% of China’s land area. By the LGM, the potential distribution of giant pandas shifted southwestward (Figure 2e), with their habitat area reduced by 28.27% to approximately 1.58 million km^2^. Most of Yunnan became suitable, while eastern regions, such as Zhejiang, Jiangsu, Jiangxi, and eastern Hunan, became unsuitable (Figure 2b). From the LGM to the MH, the potential distribution range of giant pandas expanded northeastward (Figure 2e). Regions like Jiangsu, Zhejiang, and Jiangxi became suitable again, and new suitable areas emerged in Shandong, Henan, and southern Hebei (Figure 2c). During this period, the habitat of giant pandas expanded by 75.8%, reaching approximately 2.77 million km^2^. However, from the MH to the present, the potential distribution range of giant pandas has dramatically contracted, with a 93.29% reduction in area, leaving only about 0.19 million km^2^ (Figure 2d). The distribution of giant pandas shifted northwestward during this time, with the migration distance being twice that of the LGM to MH shift (Figure 2e). Notably, the current distribution areas of giant pandas, including southern Shaanxi, central Sichuan, and southern Gansu, have remained suitable habitats across all three historical periods.

### 3.3. Impacts of Climatic Variables

During the LIG period, the mean diurnal temperature range (Bio2) had the greatest influence on the distribution of giant pandas, with a contribution rate as high as 89.8%. In the LGM period, Bio2, the mean temperature of the driest quarter (Bio9), the precipitation of the warmest quarter (Bio18), and the precipitation of the coldest quarter (Bio19) each influenced the distribution of giant pandas by more than 20%, with Bio19 having the strongest impact at 26% (Table 3). During the MH period, Bio9 and Bio18 became the primary factors influencing the distribution of giant pandas, with contribution rates of 58.1% and 40%, respectively (Table 3). In the current period, Bio9, the mean temperature of the wettest quarter (Bio8), and Bio18 are the most significant variables affecting the distribution of giant pandas, each contributing more than 20% (Table 3). Notably, isothermality (Bio3) and precipitation seasonality (Bio15) had relatively minor impacts on the distribution of giant pandas across all four periods, with contribution rates below 10%, and Bio15′s highest contribution in the current period reached just 9.2% (Table 3).

**Figure 3:**
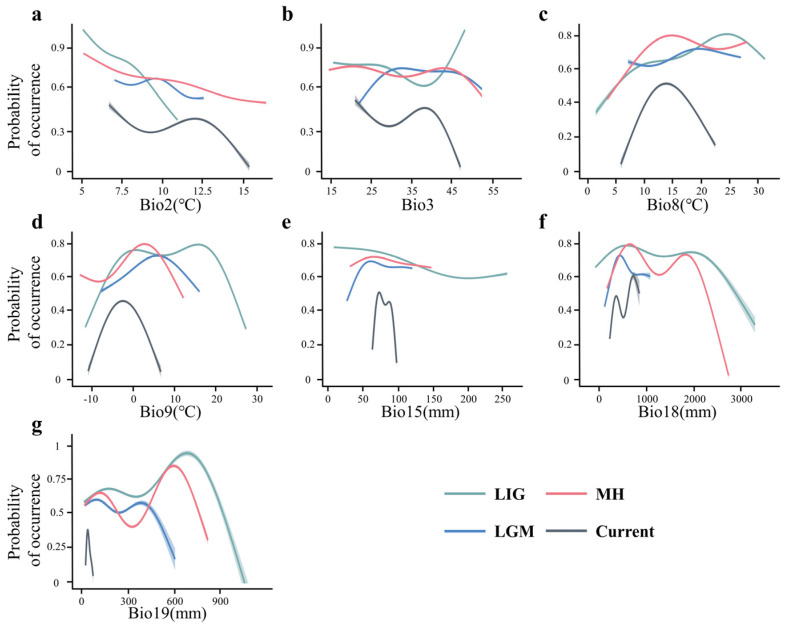
Response curves of giant pandas to seven climate variables during the Last Interglacial (green), the Last Glacial Maximum (blue), the Mid-Holocene (red), and the current period (black). (**a**) Bio2, mean diurnal temperature range; (**b**) Bio3, Iiothermality; (**c**) Bio8, mean temperature of wettest quarter; (**d**) Bio9, mean temperature of driest quarter; (**e**) Bio15,precipitation seasonality), (**f**) Bio18, precipitation of warmest quarter; (**g**) Bio19, precipitation of coldest quarter.

The response curves in Figure 3 show how the predicted occurrence probability of giant panda changes with each climatic variable. During the LIG period, giant pandas were distributed in regions with lower Bio2 values (5–7 °C), and as Bio2 increased, the probability of distribution decreased. In contrast, during the LGM period, the regions with higher probabilities of giant panda distribution had increased Bio2 values (9–11 °C), and the probability initially increased before decreasing as Bio2 continued to rise (Figure 3a). Bio2 contributed little to the models for the MH and current periods. The impact of Bio3 on giant panda distribution was relatively minor across all four periods, with no significant response curve (Figure 3b). Bio8 had a significant effect on giant panda distribution only in the current period, showing a unimodal curve with a peak slightly below 15 °C (Figure 3c). Bio9 influenced giant panda distribution during the LGM, MH, and current periods, all showing unimodal response curves. During the LGM, giant pandas were mainly distributed in regions where Bio9 ranged from 4 to 10 °C; in the MH, the distribution shifted to a smaller range of Bio9 values (6–8 °C), and by the current period, Bio9 had decreased to −4–0 °C in the distribution areas (Figure 3d). Bio15 had a minor impact only during the current period, indicating that the distribution of giant pandas was more restricted to regions with a narrower range of precipitation seasonality (70–90 mm) compared to other periods (Figure 3e). The Bio18 values in regions with higher probabilities of giant panda distribution during the LGM and current periods did not exceed 1000 mm, whereas in the MH period, Bio18 could reach up to 2300 mm (Figure 3f). Bio19 had a notable influence on giant panda distribution only during the LGM, with a higher distribution probability in regions where Bio19 ranged from 100 to 500 mm (Figure 3g).

### 3.4. Comparison of Giant Pandas’ Climatic Niches

**Figure 4:**
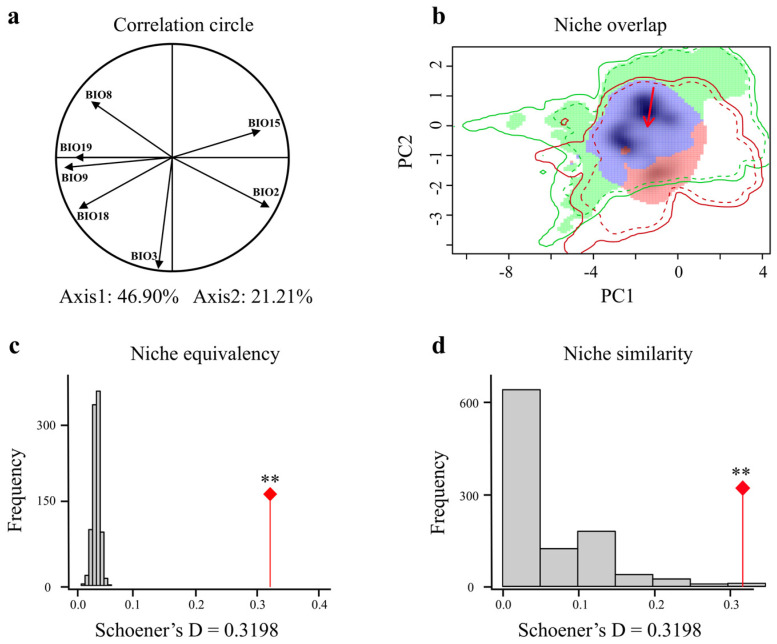
Comparison of climatic niches of giant pandas during the Last Interglacial (LIG) and Last Glacial Maximum (LGM). (**a**) Each climatic variable is described in the correlation circle along the two PCA axes. (**b**) Giant pandas in the LIG (green) and LGM (red) climatic niches, with overlapping regions in purple. Red arrows connect the centroid of niches. The outer solid line represents the climatic conditions for each period (green: LIG; red: LGM), and the dashed line represents 50% of the climatic conditions in the study area for each period. Equivalence test (**c**) and similarity test (**d**) of climatic niches of giant pandas in LIG and LGM. The red line with a diamond indicates the observed niche overlap Schoener’s D, and the gray bar indicates the simulated niche overlap. ** indicates highly significant (*p* < 0.01).

The first two axes of the climate niche space constructed from the LIG to the LGM periods explained 68.11% of the variance (Figure 4a). The climate niche of the giant panda did not reach a moderate overlap between the two periods (Schoener’s D = 0.3198), indicating that while the giant panda’s climate niche remained somewhat stable in response to climate change, certain changes also occurred. The centroid shifted along both axes 1 and 2 in a decreasing direction within the climate space (Figure 4b). The climate niche of the giant panda during the LIG and LGM periods exhibits significant similarity (*p* = 0.006) and equivalency (*p* = 0.001) (Figure 4c,d). This suggests that despite the considerable climatic differences between these two periods, the giant panda’s habitat requirements in terms of the climate have remained relatively consistent, following the principle of niche conservatism.

**Figure 5:**
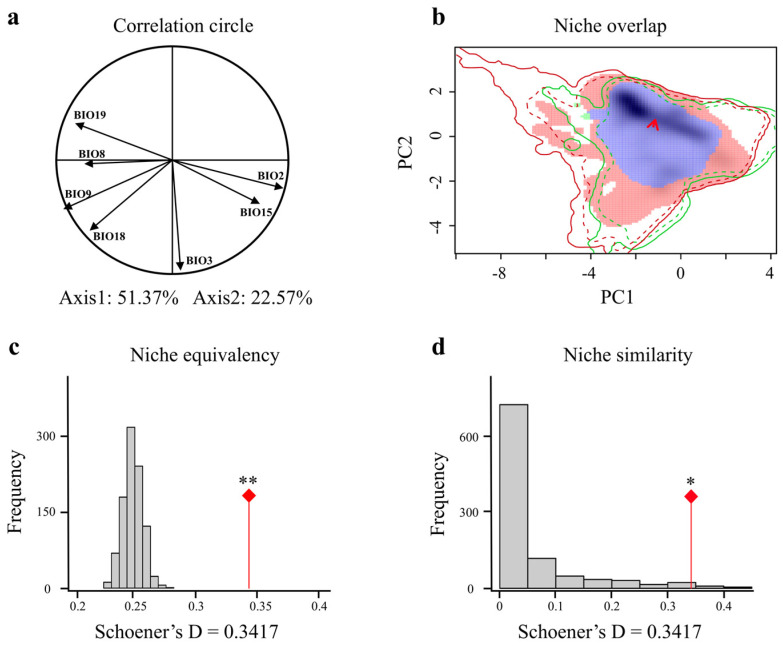
Comparison of climatic niches of giant pandas during the Last Glacial Maximum (LGM) and Mid-Holocene (MH). (**a**) Each climatic variable is described in the correlation circle along the two PCA axes. (**b**) Giant pandas in the LGM (green) and MH (red) climatic niches, with overlapping regions in purple, showing that the LGM distribution (green) is entirely encompassed within the overlapping purple area. Red arrows connect the centroid of niches. The outer solid line represents the climatic conditions for each period (green: LGM; red: MH), and the dashed line represents 50% of the climatic conditions in the study area for each period. Equivalence test (**c**) and similarity test (**d**) of climatic niches of giant pandas in the LGM and MH. The red line with a diamond indicates the observed niche overlap Schoener’s D, and the gray bar indicates the simulated niche overlap. ** indicates highly significant (*p* < 0.01), and * indicates significant (*p* < 0.05).

The first two axes of the climate niche space constructed from the LGM to the MH periods explained 73.94% of the variance (Figure 5a). The niche overlap between these two periods was slightly higher than that between the LIG and LGM (Schoener’s D = 0.3417), and the centroid exhibited almost no movement, indicating that from the LGM to the MH, the climate space of giant pandas expanded outward (Figure 5b). The niche similarity (*p* = 0.022) and equivalency (*p* = 0.001) between the LGM and MH periods are also significant (Figure 5c,d), indicating that the giant panda’s habitat climate requirements maintained a degree of continuity from the glacial period to the warmer MH, continuing to follow niche conservatism.

**Figure 6:**
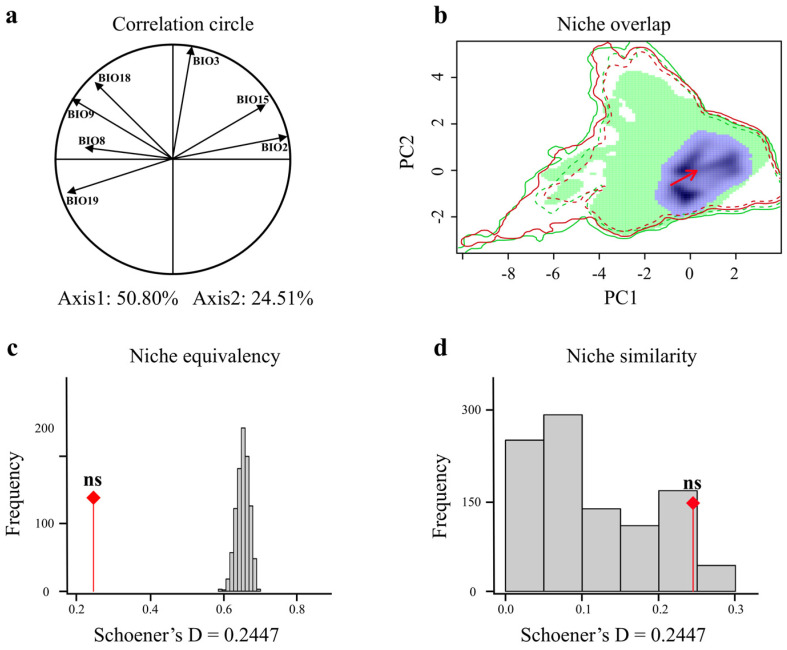
Comparison of climatic niches of giant pandas during the Mid-Holocene (MH) and current period. (**a**) Each climatic variable is described in the correlation circle along the two PCA axes. (**b**) Giant pandas in the MH (green) and current (red) climatic niches, with overlapping regions in purple, showing that the current distribution (red) is entirely encompassed within the overlapping purple area. Red arrows connect the centroid of niches. The outer solid line represents the climatic conditions for each period (green: MH; red: current), and the dashed line represents 50% of the climatic conditions in the study area for each period. Equivalence test (**c**) and similarity test (**d**) of climatic niches of giant pandas in the MH and current period. The red line with a diamond indicates the observed niche overlap Schoener’s D, and the gray bar indicates the simulated niche overlap. The ns indicates not statistically significant.

From the MH to the current period, the first two axes explained 75.31% of the variance (Figure 6a). The niche overlap between the MH and the current period was the smallest among the three time periods (Schoener’s D = 0.244), and the centroid shifted in an increasing direction along both axes 1 and 2 (Figure 6b). The similarity and equivalency tests yielded non-significant results (*p* > 0.05), indicating that there is no significant similarity or equivalency in the climate niche of the giant panda between the MH and the current period (Figure 6c,d). These findings indicate that the climate niche of giant pandas has undergone significant changes from the MH to the current period, deviating from the principle of niche conservatism.

## 4. Discussion

Existing studies emphasize the importance of integrating fossil data into ecological niche modelling, allowing historical components to be considered when estimating climate niches [92]. In this study, we collected the known fossil distribution of giant pandas and categorized them based on dating information. Using fossil distribution records and corresponding climate variables from these periods, we reconstructed the potential distribution range and climate niche of giant pandas during the LIG, LGM, MH, and current periods. Our results show that the potential distribution of giant pandas shrank southwestward and then expanded northeastward from the LIG to the MH, while their climate niche remained stable, following niche conservatism. The southwestern mountainous areas served as persistent climate refugia that remained suitable throughout all periods. However, from the MH to the current period, the potential distribution range of giant pandas significantly contracted to the northwest, and their climate niche also markedly shrank, shifting toward higher values in Bio2 and Bio15. This finding aligns with previous studies, which concluded that the climate niche occupied by mammals today differs significantly from that of the MH (before large-scale human expansion) [93]. This is due to human activities leading to a drastic reduction in the distribution range of giant pandas, both directly (through hunting) and indirectly (through habitat destruction), thereby altering their climate niche. Moreover, our findings align with previous research that compared past and current niches of giant pandas [92]. However, our study further reconstructed the climate niche of giant pandas during the MH, revealing that their climate niche expanded before shrinking from the LGM to the current, rather than undergoing a direct reduction. This finding also provides empirical evidence for large-time-scale niche conservatism [17]. The survival of giant pandas is highly dependent on a specific range of environmental conditions [43,45]. Our results can inform the development of targeted management strategies in response to future climate change. For example, giant pandas may shift their distribution to track stable climatic niches, suggesting that future reserve planning should allocate accessible areas for this potential movement. Currently, the climatic niche of the giant panda represents only a portion of its historical climatic niche. Therefore, priority conservation efforts should focus on managing habitat fragmentation and the preservation of its primary bamboo resources.

Changes in the distribution range of species before large-scale human expansion were often driven by climate change [94]. Our study found that since the LIG, the effects of climate change on giant pandas have varied across different historical periods. During the LIG and MH, the climate facilitated the expansion of the species’ potential distribution range. However, during the LGM, the climate likely restricted habitat availability, resulting in the smallest potential distribution area, which suggests that the LGM served as a population bottleneck for giant pandas. This is consistent with previous interpretations [35], and similar climate-driven habitat contractions have been observed in other large mammals, such as tigers [28], indicating that glacial periods had widespread effects on species distributions. Since the MH to the current period, humans have extensively modified natural landscapes, with urban and agricultural expansion encroaching upon the most fertile and habitable terrestrial environments, fundamentally altering global ecosystems [93]. The giant panda’s potential distribution range experienced a significant contraction during this period. While climate change has undoubtedly played a role in shaping the panda’s distribution, the extent to which it contributed to the substantial habitat reduction from the MH to the current period remains unclear. Determining the specific contribution of climatic factors to species distribution changes is challenging, but advances in fossil records and modelling techniques may provide a deeper understanding of climate–species interactions.

The mean diurnal temperature range (Bio2) had a significant influence on the potential distribution of giant pandas during both the LIG and LGM periods, with a particularly high contribution rate of 89.8% during the LIG. This suggests that in the warmer interglacial period, giant pandas likely preferred regions with smaller diurnal temperature ranges, which may be linked to their need for a stable temperature environment. However, as the climate shifted into the LGM period, the effect of the diurnal temperature range on giant panda distribution decreased, possibly reflecting the species’ adaptation to colder climatic conditions. The mean temperature of the driest quarter (Bio9) has consistently impacted the distribution of giant pandas across multiple periods, especially during the LGM, MH, and current periods. This indicates that the potential distribution of giant pandas is highly sensitive to the temperature of the driest season. During the LGM, giant pandas tended to inhabit warmer dry-season regions (4–10 °C), while in the current period, the dry-season temperatures in their distribution areas have significantly decreased (−4–0 °C). This shift may be closely related to cooling climates and the associated changes in vegetation and habitat structure. Temperature changes could affect the availability of food resources, such as bamboo, which may experience growth and reproduction limitations during cold and dry periods [95]. Furthermore, we can identify potential future habitats based on the sensitivity of giant pandas to temperature changes in order to develop effective conservation strategies.

The mean temperature of the wettest quarter (Bio8) and the precipitation of the warmest quarter (Bio18) have also shown significant influence on giant panda distribution in the current period. The peak value of Bio8, around 15 °C, suggests that giant pandas prefer mild temperatures in humid climates. This is closely associated with the growth conditions of bamboo, their primary food source, which thrives in warm and moist environments, providing an abundant food supply for giant pandas [95,96]. Similarly, Bio18 reflects that giant pandas are distributed in regions with high precipitation during the warmest season, highlighting their habitat’s dependence on sufficient rainfall. Precipitation has a notable impact on the vegetation in giant panda habitats, particularly during warm seasons. Existing studies indicate that precipitation significantly influences the dynamics of giant panda distribution but is negatively correlated with their range expansion [97]. Therefore, further research is needed to better understand how variations in precipitation create different microenvironments and the resulting fine-scale effects on panda distribution. The precipitation of the coldest quarter (Bio19) had a notable impact on giant panda distribution during the LGM, reflecting their dependence on cold-season precipitation during glacial periods. As glaciers expanded, the combination of colder climates and a lower precipitation likely forced giant pandas to shift to areas that still received substantial cold-season precipitation.

On the other hand, isothermality (Bio3) and precipitation seasonality (Bio15) had relatively minor effects on giant panda distribution, with Bio3 showing no significant response curves across all periods. This suggests that giant pandas exhibit low sensitivity to isothermality, relying more on absolute temperature values and seasonal changes rather than the consistency of temperature fluctuations. Bio15 only had a moderate influence on giant panda distribution in the current period, potentially linked to increased climate variability and unstable precipitation patterns. This indicates that giant pandas may prefer regions with relatively stable precipitation seasonality, which aligns with current research [7,98,99,100]. The shifts in these climatic factors across different historical periods highlight the adaptability of giant pandas to changes in their habitat. Understanding these relationships provides critical insights into how climate changes have influenced giant panda distribution over time and will be essential for predicting their future habitat suitability under ongoing climate change.

The climate niche analysis reveals the variations in the giant panda’s climatic niche across different historical periods. From the LIG to the LGM, the niche overlap was low, but the similarity and equivalency tests were significant. This suggests that despite the climate transition from a warm interglacial to a colder glacial period, the giant panda’s climatic niche remained generally stable, reflecting niche conservatism. Niche conservatism on larger time scales is mostly observed in plants [101], and we report for the first time the niche conservatism of giant pandas on large time scales. This also confirms the conclusion of previous study that animals and plants have the same pattern in the evolution of climatic niches [102]. From the LGM to the MH, the panda’s climatic niche expanded, likely due to the retreat of glaciers and the resulting increase in suitable habitat areas. This was especially true in warmer and more humid conditions, where bamboo, the panda’s primary food resource, became more abundant. The similarity and equivalency tests remained significant, indicating that the giant panda’s climatic requirements continued to persist during this period, further demonstrating niche conservatism. This suggests that giant pandas can adjust their distribution range to track stable climate niches, highlighting the need to allocate accessible areas in future conservation planning and regularly monitor and adjust priority conservation zones as necessary.

However, from the MH to the current period, the giant panda’s climatic niche underwent significant changes, no longer aligning with past niche patterns. This shift likely reflects the influence of multiple factors, including changes in temperature and precipitation patterns, as well as human activities, and giant pandas gradually migrated to mountainous areas, completing the adjustment of the climatic niche, which is consistent with a previous study [35]. Habitat fragmentation and human interference in panda habitats may have forced the species to adapt to new climatic conditions, even altering their traditional habitat preferences. The current realized climatic niche of the giant panda represents only a portion of its fundamental niche, suggesting that climate change may indirectly impact pandas by affecting their primary bamboo sources (e.g., *Fargesia qinlingensis*), rather than exerting direct pressure on the species. Thus, managing habitat fragmentation and changes in land use will remain priority actions for future conservation efforts.

We utilized the MaxEnt model to infer the range of giant pandas during the target period and employed the ecological niche comparison framework [90], both of which have been demonstrated to be reliable [101,103,104,105]. However, there remain several sources of uncertainty in this study that may potentially impact some of the results. During the period from the LIG to the MH, in addition to climate change, biotic interactions may have also facilitated the range shift of the giant panda. However, due to a lack of data, this aspect was not considered in our study. The fossil localities used for modelling during the target period may not have been exhaustive, and while the powerful predictive ability of the MaxEnt model compensates for this limitation, if multiple new fossil sites are discovered beyond the predicted range during the target period, our model would need to be revised. Furthermore, this study implies a presumption that the individuals represented by the fossils succumbed to natural death rather than being influenced by climate change. If this assumption is unfounded, the accuracy of the present research would be called into question, highlighting an inherent limitation in fossil record studies of ancient events that is challenging to circumvent. The climate during geological periods oscillates, and our study simplified this process. A more refined time scale would expand our understanding of the impact of paleoclimatic changes on species. Nevertheless, our study enriches our understanding of how large mammals respond to paleoclimatic changes and will provide insights for the development of conservation strategies for giant pandas under the background of climate warming.

## 5. Conclusions

Our conclusion is that from the Late Pleistocene to the period before large-scale human modification of the environment, the potential distribution range of the giant panda underwent significant changes, initially contracting and then expanding, while consistently maintaining a stable climate niche, adhering to the principle of niche conservatism. This indicates that the giant panda has the ability to shift its distribution to track stable climate niches. Therefore, future conservation planning should designate accessible areas for the giant panda and regularly monitor and adjust priority conservation areas as needed. Since the MH, the giant panda has gradually retreated to its current distribution range. The climatic niche currently occupied by the giant panda is only a small fraction of its historical climate niche. This suggests that the giant panda may have the potential to cope with future climate change, which could indirectly impact the species rather than directly affect it. Therefore, priority conservation efforts should focus on managing habitat fragmentation and the preservation of its primary bamboo resources. Additionally, it is recommended to incorporate climate models into conservation planning to better predict the potential impacts of future environmental changes on giant panda habitats, thus developing more effective conservation strategies.

## Figures and Tables

**Table 1:** List of 19 climate variables used in the model development.

Code	Description	Unit
Bio1	Annual mean temperature	°C
Bio2 *	Mean diurnal temperature range	°C
Bio3 *	Isothermality	−
Bio4	Temperature seasonality	−
Bio5	Max temperature of warmest month	°C
Bio6	Min temperature of coldest month	°C
Bio7	Temperature annual range	°C
Bio8 *	Mean temperature of wettest quarter	°C
Bio9 *	Mean temperature of driest quarter	°C
Bio10	Mean temperature of warmest quarter	°C
Bio11	Mean temperature of coldest quarter	°C
Bio12	Annual precipitation	mm
Bio13	Precipitation of wettest month	mm
Bio14	Precipitation of driest month	mm
Bio15 *	Precipitation seasonality	−
Bio16	Precipitation of wettest quarter	mm
Bio17	Precipitation of driest quarter	mm
Bio18 *	Precipitation of warmest quarter	mm
Bio19 *	Precipitation of coldest quarter	mm

Note: the variables selected for analysis are marked with an asterisk (*).

**Table 2:** Evaluation metrics of MaxEnt models for giant pandas across four temporal periods using ENMeval.

Target Period	Model	FC	RM	AUC_TEST_	AUC_DIFF_	OR_10_	ΔAICc	MaxSSS
LIG	Default	LQHPT	1	0.928	0.026	0.330	27.107	−
	Optimized	LQHP	3.5	0.917	0.010	0.077	0	0.4758
LGM	Default	LQHPT	1	0.957	0.018	0.205	82.820	−
	Optimized	LQH	2	0.961	0.017	0.170	0	0.5194
MH	Default	LQHPT	1	0.893	0.062	0.4	NA	−
	Optimized	LQH	4	0.890	0.044	0.3	0	0.5240
Current	Default	LQHPT	1	0.998	0.001	0.122	74.249	−
	Optimized	LQH	0.5	0.998	0.001	0.129	0	0.1249

Notes: AUC_TEST_ refers to area under the receiver–operating characteristic curve for test localities; AUC_DIFF_ refers to training–testing AUC difference; OR_10_ refers to 10% omission rate for test localities; ΔAICc refers to difference in corrected Akaike information criterion.

**Table 3:** Contribution rates of bioclimatic variables to the MaxEnt model of giant pandas across different climate periods.

Variables	Description	LIG (%)	LGM (%)	MH (%)	Current (%)
Bio2	Mean diurnal temperature range	**89.8**	22.6	1.7	0.9
Bio3	Isothermality	1.3	6.6	0	6.6
Bio8	Mean temperature of wettest quarter	8.7	2.5	0	24.1
Bio9	Mean temperature of driest quarter	4	21.3	**58.1**	**27.5**
Bio15	Precipitation seasonality	0	1	0	9.2
Bio18	Precipitation of warmest quarter	0	20	40	22.8
Bio19	Precipitation of coldest quarter	4.8	**26**	0.2	8.9

Note: the most important variables are shown in bold.

## Data Availability

No new data were created.
